# COLLEGE EDUCATION AND LEISURE ACTIVITIES PREDICT LATE-LIFE COGNITIVE STATUS: DO BIRTH COHORTS DIFFER?

**DOI:** 10.1093/geroni/igae098.3309

**Published:** 2024-12-31

**Authors:** Jacqui Smith, Kimson Johnson, Marina Larkina

**Affiliations:** University of Michigan, Ann Arbor, Michigan, United States; University of Michigan, Ann Arbor, Michigan, United States; University of Michigan, Ann Arbor, Michigan, United States

## Abstract

Efforts to determine potentially modifiable factors associated with heterogeneity in cognitive status in old age point to the importance of participation in cognitive, physical, and social leisure activities and the relevance of historical changes in completion of post-secondary education. We use data from two birth cohorts in the US Health and Retirement [HRS: Baby Boomers (BB) born 1948-1959; Older cohorts (OC) born before 1948] to examine this proposal. Eligible participants (N=8503: Mage=71) provided post-secondary education information in the HRS Life History Mail Survey (LHMS, 2015-2017) and cognitive status and participation in leisure activities in the 2016 or 2018 HRS biennial waves. Cohorts differed in rates of college education (BB 53% vs OC 42%) and overall leisure activity participation (BB>OC, p<.001). A multiple regression analysis predicting cognitive status (normal vs impaired: Langa-Weir algorithm) revealed significant effects for college education (OR=O.47), leisure activities (OR=0.87), and cohort (ps <.001), after controls for age, gender, wealth, race, marital status, chronic illnesses, and depression. In follow-up cohort-stratified analyses, we grouped people by level of participation in specific activity types (top quartile vs rest). Interestingly, within the older cohort, the risk of cognitive impairment was significantly reduced by high participation in cognitive (OR=0.42) and physical (OR=0.71) leisure activities in addition to having college education (OR=0.47). Within the BB cohort, only people with high participation in cognitive leisure activities (OR=0.65) in addition to college education (OR=0.38) had a reduced risk. Future research should investigate why some leisure activities seem to confer benefits for cognitive resilience whereas others don’t.

